# Supplementary material for: Mapping Evolution of Molecules across Biochemistry with Assembly Theory
Source: J Chem Inf Model. 2026 Jul 7;66(14):8239–50. doi: 10.1021/acs.jcim.6c00939 (PMC13417883; doi:10.1021/acs.jcim.6c00939)
Supplement: Supplementary file 1 [file ci6c00939_si_001.pdf]

Supporting information for

Mapping Evolution of Molecules Across Biochemistry with Assembly Theory

Sebastian Pagel, Abhishek Sharma, and Leroy Cronin*

School of Chemistry, The University of Glasgow, University Avenue, Glasgow G12 8QQ, UK, *Corresponding author email: Lee.Cronin@glasgow.ac.uk

Contents

1 Assembly Pathways 4

1.1 Assembly Pathway Calculations 4

2 Joint Assembly Spaces 4

2.1 Construction of Joint Assembly Spaces 4

2.2 JAS of Natural Products (Assembly Observed) 5

2.3 JAS of PubChem Molecules (Assembly Possible) 5

2.4 JAS of Drug Molecules 5

2.5 JAS Visualisations 5

3 Contingency Loss Description 9

3.1 Contingency Loss by Assembly Depth 9

3.2 Contingency Loss by Pathways 10

4 Molecule Generation Pipeline 12

4.1 Algorithm Description 12

4.2 Fragment Sampling 13

4.3 Selectivity Scaling 14

4.4 Number of bonds per construction step 14

4.5 Supported Element Types 15

5 Molecule Filter Description 16

5.1 Disallowed Substructure Filter 16

5.2 Conformer Generation and Geometry Optimization 18

5.3 Distorted Angle and Bond Filter 18

5.3.1 Database construction of bond and angle-parameter distributions 19

5.3.2 Gaussian Mixture Models of Bond and Angle Parameters 19

5.3.4 Filtering of unusual bond- and angle parameters 21

5.4 Disallowed Substructure Filter Examples 22

5.5 Conformer and Geometry Optimization Examples 23

5.6 Distorted Bond and Angle Filter Examples 24

6 Divergence estimation 25

6.1 Molecular Similarity Quantification 25

6.2 JAS Similarity Quantification 25

7 Influence of Molecule Generation Hyperparameters 25

7.1. Influence of Adjusting Sampling Weights for Assembly Depth Selection 25

7.2 Influence of Adjusting Sampling Weights for Fragment Selection 27

7.3 Influence of adjusting sampling weights for the number of construction steps selection 28

8 Reconstruction of molecules from partial Contingency 30
8.1 Reconstruction of molecules for the estimation of divergence 30
8.2 Reconstruction of molecules for the estimation of the exploration rates 31
8.3 Reconstruction of molecules for the generation of drug-like molecules 31
8.4 Reconstruction of molecules from the assembly pathway of Brefelamide 31
9 Database Analysis 32
9.1 Number of Bonds Distribution in PubChem and COCONUT Databases 32
9.2 Object Distributions in PubChem and COCONUT Databases 33
9.3 Object Utilization in the JAS of COCONUT and PubChem 35
10 Quantification of exploration in Joint Assembly Spaces 36
10.1 Exploration rate of JAS_{PC} by JAS_{C} considering only COCONUT building blocks 37
11. Analysis of lost molecular structures 38
References 39

1 Assembly Pathways

1.1 Assembly Pathway Calculations

Assembly pathways were either calculated by the algorithm described in Jirasek *et. al.*[1] written in the Go programming language, or an updated version of the algorithm written in C++, which is conceptually identical but optimised for computational efficiency (to be published). Calculations with the algorithm described in Jirasek *et. al.* were performed with a timeout of 10 minutes and 10 workers, whereas calculations with the second algorithm were performed with a timeout of 2 minutes.

After calculating the assembly pathways and assembly indexes, the assembly depth was computed using a recursive approach. Building blocks (i.e. two atoms connected by a bond in this case) were assigned an assembly depth of 0, and each subsequent fragment the maximum assembly depth of its parents + 1.

2 Joint Assembly Spaces

2.1 Construction of Joint Assembly Spaces

Since the exact calculation of the Joint Assembly Space (JAS) of a large set of molecules poses a computationally almost intractable challenge, the JAS in this study was approximated by taking the union of individual molecules assembly pathway. To do so first, the assembly pathways of a set of molecules were calculated as described above. Then, the individual pathways were combined using an adapted version of the *compose_all* function from the *Python* library *NetworkX* (version 2.8.8) into a directed multigraph (MultiDiGraph). During this step, each unique node was assigned a set of features. The *level*-attribute was set to correspond to the lowest assembly depth (*d*) found among all considered pathways. Additionally, the *count* attribute represents the number of pathways in which a fragment or node appeared whereas the *usage* attribute contained a list of the objects' assembly depths which were constructed using this node. Additionally, Morgan fingerprints (size 512, radius 2) were calculated using the Python library RDKit[2,3]. To help with the efficient selection of compatible fragments, atoms

with free valence were stored under the *atomic_count* attribute (see Molecule Generation Pipeline). All molecules were pre-processed using RDKit to remove all non-covalently bound parts.

2.2 JAS of Natural Products (Assembly Observed)

The JAS of Natural Products was calculated from the assembly pathways of 211731 molecules from the COCONUT database with a maximal assembly depth of 20 (see above). A subsample of these assembly pathways was used to calculate and benchmark various parts of the algorithms described in this work as indicated.

For the quantification of selection in Earth's chemical space of natural products, all molecules with an assembly depth of up to 25 were considered.

2.3 JAS of PubChem Molecules (Assembly Possible)

The JAS of more than 70 million molecules from the PubChem database was estimated. All molecules were sorted by the number of bonds and all duplicate structures were removed. The assembly pathways of all molecules with up to 32 bonds (between heavy atoms only) were then calculated as described above, and the assembly depth was calculated for all fragments.

2.4 JAS of Drug Molecules

10656 small molecules with a maximal assembly depth of 20 from the ChEMBL database were used to construct the JAS of drug molecules. The molecules were pre-processed and filtered using RDKIT. All hydrogens were removed, and any non-covalently bound parts were removed. The assembly pathways were calculated using the C++ algorithm, and the JAS was constructed from the pathways as described above.

2.5 JAS Visualisations

Exemplary JAS are presented in an assembly depth ordered, layered graph throughout the manuscript and supplementary information. Because of the complexity of the JAS of a large set of molecules, only

examples of a subset of molecules are presented. In general, all graphs are organized with objects of assembly depth 0 (building blocks) in the lowest layer, and all subsequent layers correspond to objects (contingent or observed) of increasing assembly depth. Unless a contingency loss or single pathway is highlighted, building blocks and contingent objects are represented as green nodes, with diameter and colour intensity indicating the number of pathways a given object has appeared in. Observed objects are represented as red nodes in this case. Likewise, edges between nodes as scaled by width and colour intensity. An exemplary JAS from 24 randomly sampled molecules (Supplementary fig. 1) is shown in Supplementary fig. 2.

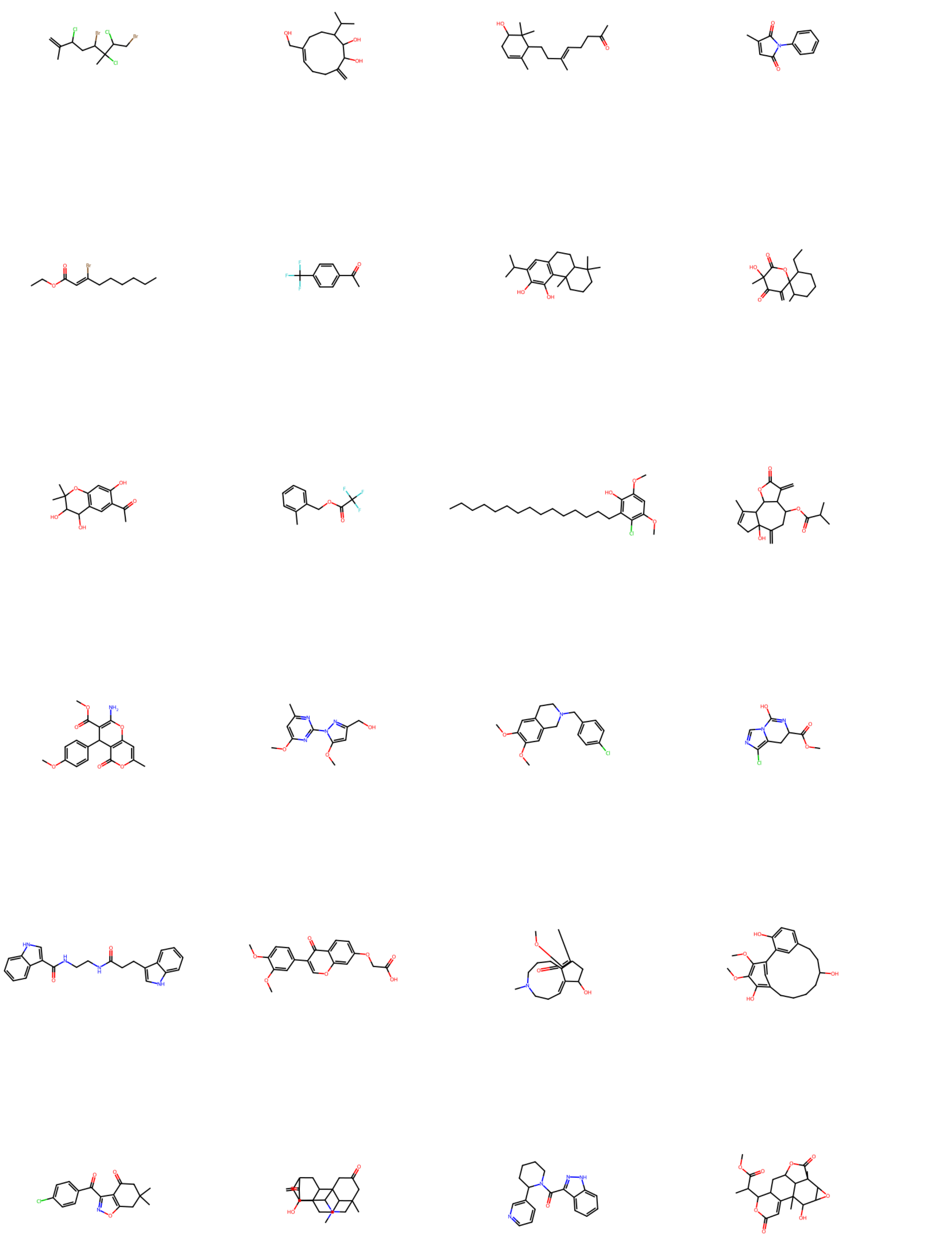

Supplementary figure 1: **The set of 24 molecules that were used to generate the JAS depicted in Supplementary figures 2–5.** The molecules were randomly sampled from the COCONUT database of molecules with a maximal assembly depth of 14.

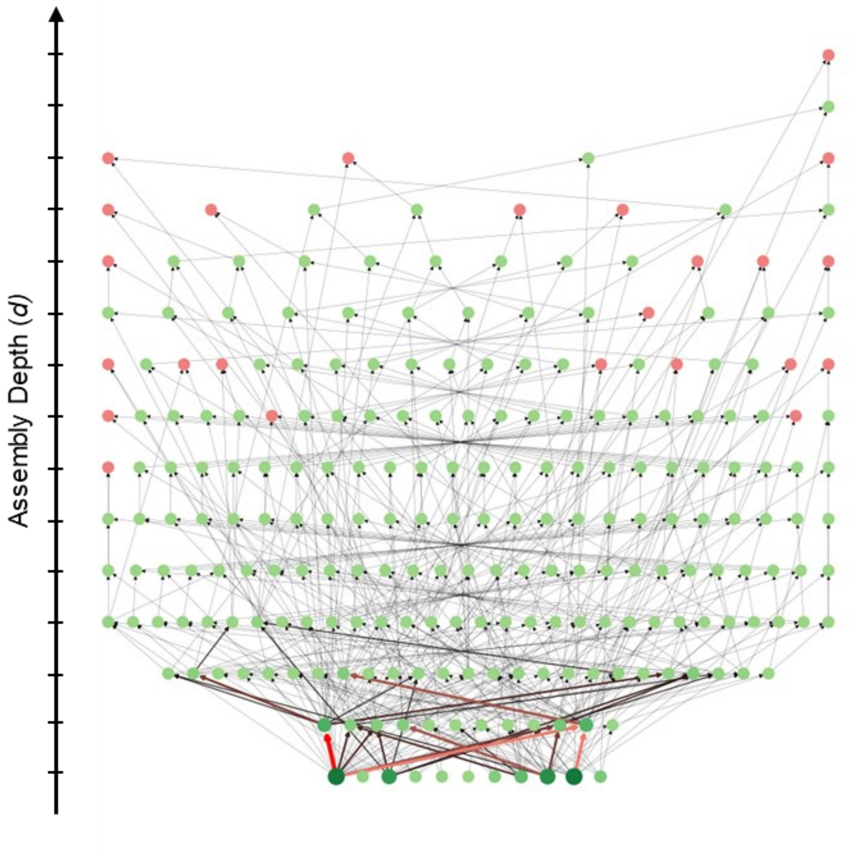

Supplementary figure 2: Exemplary depiction of a JAS. The JAS of 24 randomly sampled molecules from the COCONUT database with a maximal assembly depth of 14 was constructed from their assembly pathways. The assembly pathways were combined into a single JAS by taking the union of their graphs as described in Section 2.1. Observed objects are depicted as red nodes, whereas building blocks and contingent objects are shown in green. The size as well as the intensity of their colour is scaled proportional to the number of times objects occurred in the individual pathways. Likewise, the thickness and colour of edges are scaled proportional to the number of times one object was used to construct a respective other object.

In case a molecule pathway is highlighted, the colour intensity for nodes and edges scaled by their importance (proportional to the number of pathways they have occurred in) is removed. Nodes that correspond to the given highlighted pathway are coloured in dark green (see Supplementary fig. 3).

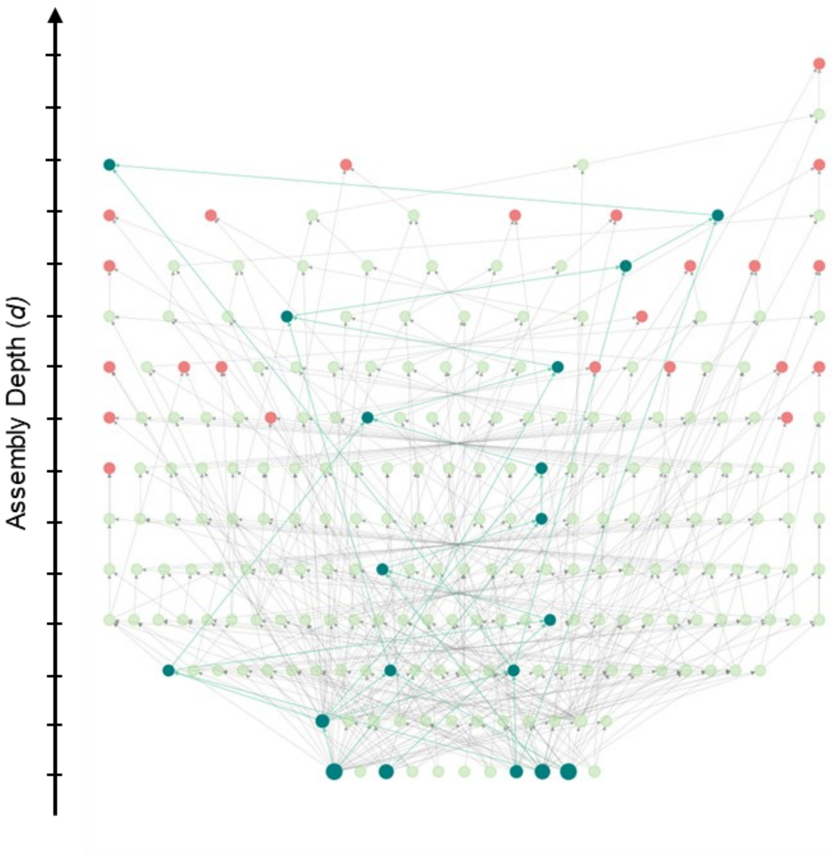

Supplementary figure 3: Exemplary depiction of a JAS. 24 molecules from the COCONUT database with a maximal assembly depth of 14 were randomly sampled and their JAS was constructed from their assembly pathways as described in section 2.1. One molecule assembly pathway within this JAS is highlighted in dark green. All other objects (building blocks, and contingent objects) are coloured in light green. Observed objects are shown as red nodes. The size as well as the intensity of their colour is scaled proportional to the number of times objects occurred in the individual pathways.

3 Contingency Loss Description

To induce a contingency loss given a JAS either objects with an assembly depth larger than a given threshold were removed, or the entire pathway that corresponds to that object, given the objects on that pathway do not occur in any other remaining objects pathway as will be described in the following.

3.1 Contingency Loss by Assembly Depth

Contingency loss by loss of objects with an assembly depth larger or equal to a given assembly depth d_{max} all objects (observed or contingent) with d_{max} or larger are removed from the JAS. An

illustrative example is given in Supplementary fig. 4 below. All other objects, be they only present on pathways from removed higher objects or not, remain unchanged.

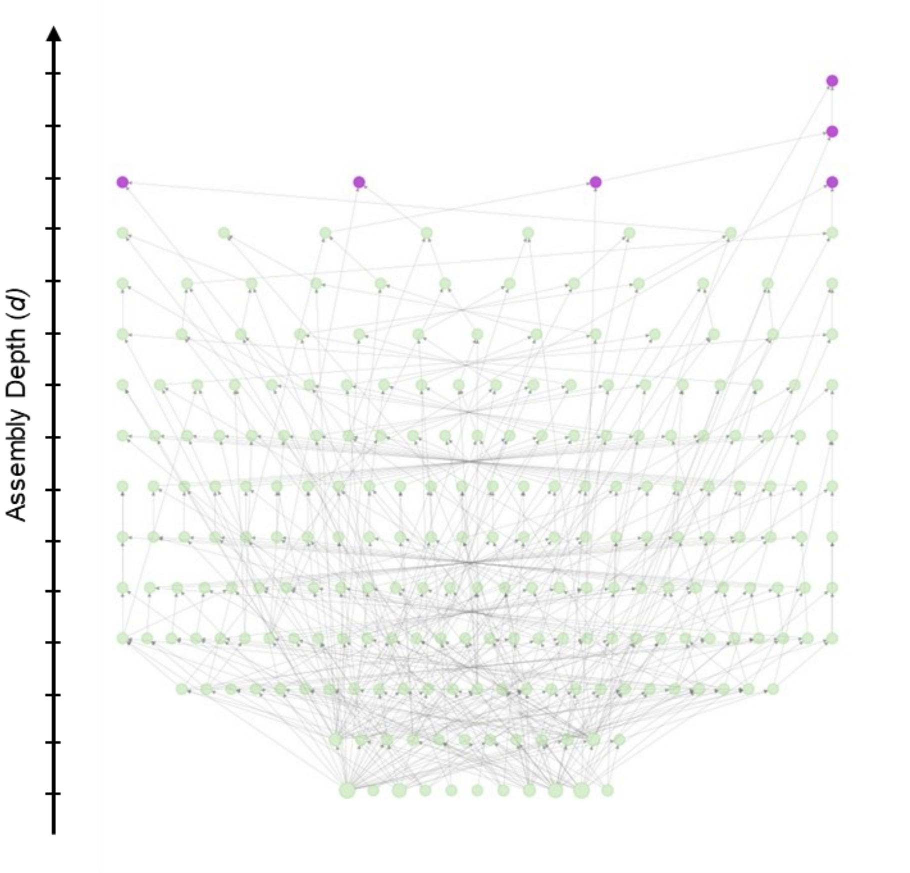

Supplementary figure 4: Exemplary depiction of contingency loss by assembly depth. The JAS of 24 randomly sampled molecules from the COCONUT database with a maximal assembly depth of 14 was constructed as described in section 2.1. Contingency was removed from this JAS by removing objects with an assembly depth of 12 or above. Removed objects are shown in purple. The remaining objects are shown as light green nodes. The size as well as the intensity of their colour is scaled proportional to the number of times objects occurred in the individual pathways.

3.2 Contingency Loss by Pathways

Loss of entire pathways instead of objects with an assembly depth of more than a certain threshold was performed by first, removing all observed objects with an assembly depth larger than d_{max}. Then, objects that were only present on those observed objects pathways were also removed. All objects that were part of an observed objects pathway, but are also present on at least one remaining object's pathway were not removed. The *count* attribute of the respective object in the remaining JAS was

reduced by one for each observed object's pathway that was removed and a given object was on. An illustrative example is given in Supplementary fig. 5 below.

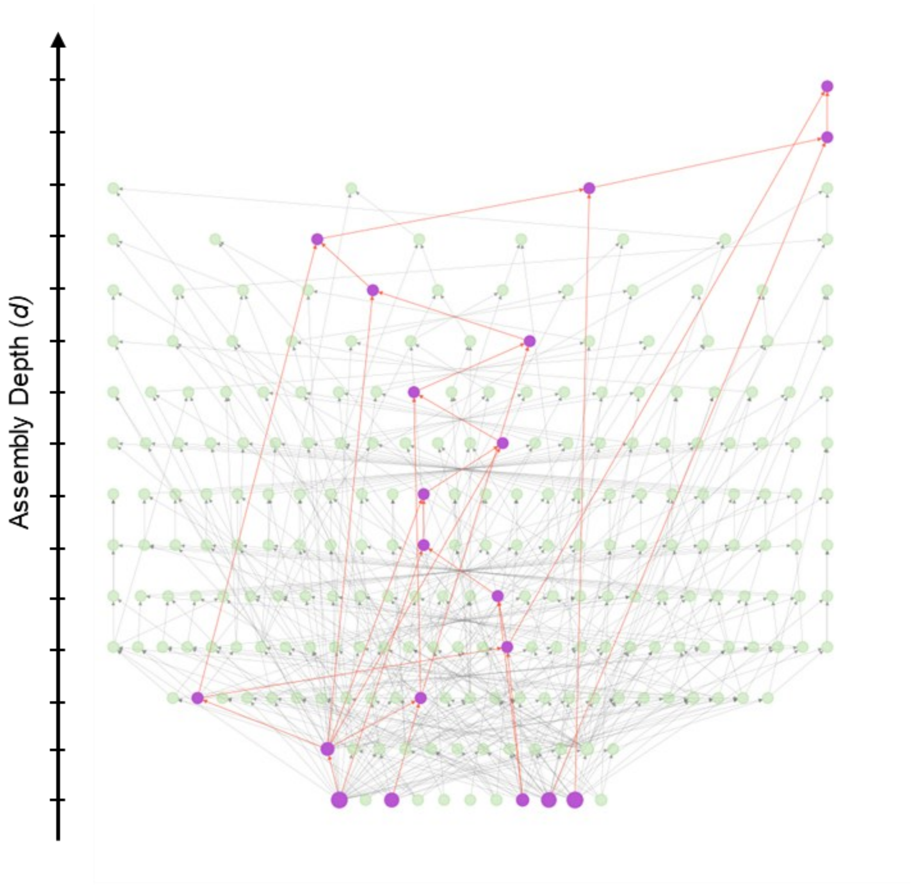

Supplementary figure 5: Exemplary depiction of contingency loss by removing pathways. The JAS of 24 randomly sampled molecules from the COCONUT database with a maximal assembly depth of 14 was constructed as described in section 2.1. Contingency was removed by removing an observed object and objects exclusively occurring on its assembly pathway. The pathway of the removed object is highlighted in purple. The remaining objects are shown as light green nodes.

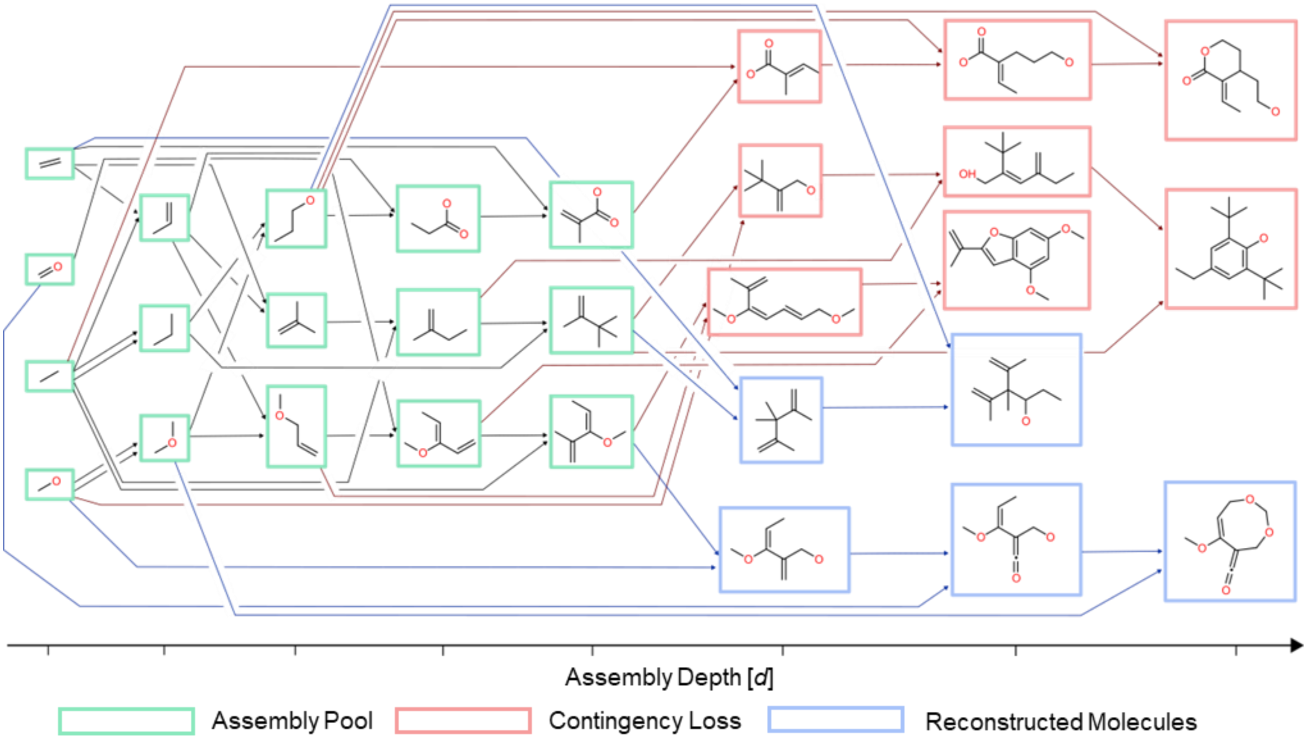

Supplementary figure 6: Exemplary JAS of three molecules, contingency loss, and reconstruction. The JAS of three molecules that were randomly sampled from the COCONUT database was constructed as described in section 2.1 (green and red boxes). After a contingency loss of $\omega = 3$ (red boxes) the available assembly fragments (green boxes) were used to reconstruct new molecules (blue boxes). The reconstruction algorithm is described in detail in the section 4.

4 Molecule Generation Pipeline

4.1 Algorithm Description

New molecules, given a set of building blocks or a JAS were constructed via algorithms described in Fig. 2 (main text). Here a detailed description of these algorithms is provided. Given a JAS (e.g. of natural products), new molecules are typically reconstructed by first (optionally) removing a section of this JAS, either by removing fragments with an assembly depth larger than some maximal assembly depth d_{max}, or these fragments and objects on their pathways (contingent objects). Contingent objects that appear exclusively on the pathway of a molecule that was removed will also be deleted and not available for subsequent constructions of new molecules. In case the contingent objects appear on more than one pathway. The object in the truncated JAS defined as JAS_{dmax} is then utilized to construct new

molecules in the following way. First, the weights to sample a fragment from the JAS are set according to the out-degree of a fragment's node in the JAS_{dmax}. Then, the sampling weights for the number of construction steps are set by the number of observed objects per assembly depth. To start the generation of a molecule, the number of construction steps is sampled as well as an initial fragment (f_1) from the top level of the maximal assembly depth in JAS_{dmax}. Next, for each step in the construction process, an assembly depth to select a fragment from is sampled, and a fragment from that assembly depth, f_2 (for more details see below Fragment Sampling). The sampling is optionally scaled or adjusted by some selectivity factor (refer to Selectivity Scaling for more details). f_1 and f_2 are then combined to form a new object by overlapping atoms of the same element type and enough free valence to contain all bonds from both fragments without valence violations in the subsequently generated fragment. The number of atoms to be overlapped is sampled according to the distribution of overlaps found in the construction of the JAS of natural products. This process is repeated until the sampled number of construction steps have been completed. At this point, the constructed molecules are optionally filtered as described in the molecule filtering section below. After each construction the new contingent and observed objects are added to the JAS and the sampling weights are adjusted accordingly.

In the case that molecule construction does not start from a JAS, but from a set of building blocks the construction process slightly changes. Instead of weighting the selection of new fragments by the out-degree of the corresponding node in the graph of the JAS, the selection is performed randomly. As the JAS is being constructed, the sampling weights can optionally be adjusted as described above.

4.2 Fragment Sampling

In the assembly process described in Jirasek *et. al.*[1] molecules are assembled by overlapping *n* atoms between two molecules (or molecular fragments), ensuring that the resulting molecule does not violate any valence rules. To efficiently select fragments that can be combined to form a valid molecule (by valence rules), elements with free valence are stored as node features for each fragment (building blocks, contingent objects, and observed objects) in the JAS. This allows us to rapidly test if two

fragments are likely to be allowed to be combined in an assembly process compatible way via a simple Boolean *AND* operation. While not being perfectly immune to false positives, this prefiltering step greatly increases the chance of selecting a pair of two fragments that can combined into a valid molecule. In case a false positive fragment was sampled in a construction step, this fragment is removed from the set of available fragments for this construction step.

4.3 Selectivity Scaling

A selectivity factor is introduced by scaling the sampling weights for the fragment selection per construction step. In the simplest case, this is done by scaling the out-degree of respective nodes corresponding to fragments in the JAS with an exponential factor. In the case of the JAS of natural products, setting this factor to 0, intuitively corresponds to uniformly sampling over available fragments to construct a new molecule, while setting this selectivity factor equal to 1 mimics the selectivity of the JAS of natural products during a construction step. While the selectivity factor can simply describe the transition from a uniform or random selection to mimicking the natural product JAS, it can easily be adjusted to be more chemically informed. This includes the prevalence on a set of functional groups (e.g. for toxicologically relevant functional groups), element compositions or any other descriptor.

4.4 Number of bonds per construction step

As mentioned above, Assembly Theory on molecules constructs molecules from building blocks by combining two fragments on a number of atoms of the same element type. Theoretically, the number of atoms on which two fragments are combined is only limited by the number of atoms with sufficient free valence available in these two fragments. The only other limitation is that no two atoms from the same fragment, cannot selected if they are connected by a bond already. To accurately mimic the assembly process the JAS of 10k randomly selected molecules from the COCONUT database was constructed as described above to get a distribution of the number of combined atoms per assembly

construction step. This distribution is employed in the Algorithm described above to sample on how many atoms two fragments are to be connected on.

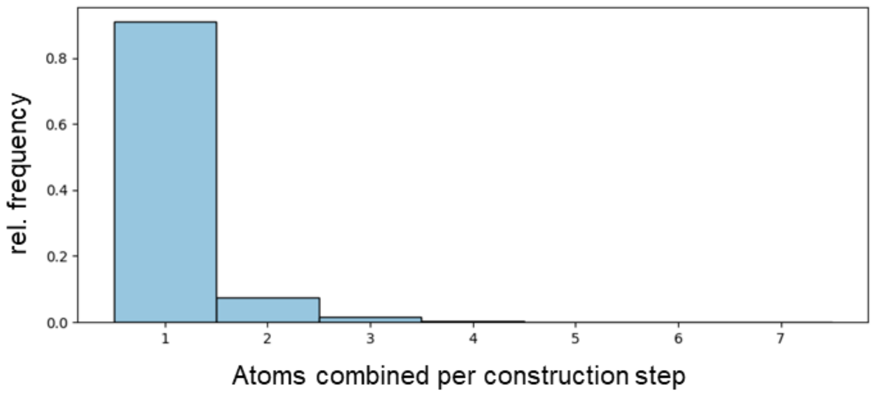

Supplementary figure 7: Distribution of the number of atoms combined per assembly step. The JAS of 10000 randomly sampled molecules from the COCONUT database was constructed as described in section 2.1. From this JAS the frequency of the number of atoms combined per construction step was then calculated.

4.5 Supported Element Types

The construction of molecules was limited to the elements with their respective maximal valence according to Supplementary table 1.

Supplementary table 1: Allowed element types and their maximum valence in the molecule construction

Element Type	Maximum valence
C	4
N	3
O	2
F	1
P	5
S	6
Cl	1
Br	1
I	1
Si	4
As	5
Se	2
B	3

5 Molecule Filter Description

The filtering pipeline consists of structural filtering steps, in which similar structural filters are being applied as in MOLGEN software[4]. In the second step, *n* conformers of the molecules passing the structural filter are generated with RDKit and subsequently optimized with the UFF forcefield[2,5]. Molecules pass this filtering step if any of the *n* generated conformers is being successfully optimized. The conformers resulting in passing this filtering step, are then checked for unusual bond- and angle parameters. Molecules which's conformers have more than a cut-off number of bad bond- and angle parameters, are being discarded.

5.1 Disallowed Substructure Filter

Substructure filters are typically used to filter unfeasible molecules. Here we adapted the filters implemented in MOLGEN[4] listed in Supplementary table 2. These filters mostly target molecules with unfavourable ring strains. An additional substructures was added to filter out molecules with intrinsically unstable cyclobutadiene-like motifs.

Supplementary table 2: Disallowed substructures adapted from MOLGEN[4]

Filter Number	SMARTS pattern
1	[!#1]1:[!#1]:[!#1]:[!#1]2:[!#1](:[!#1]:1)~[!#1]~2
2	[!#1]1:[!#1]:[!#1]:[!#1]2:[!#1](:[!#1]:1)~[!#1]~[!#1]~2
3	[!#1]1:[!#1]:[!#1]2:[!#1]:[!#1](:[!#1]:1)~[!#1]~2
4	[!#1]1:[!#1]:[!#1]2:[!#1]:[!#1](:[!#1]:1)~[!#1]~[!#1]~2
5	[!#1]1:[!#1]:[!#1]2:[!#1]:[!#1](:[!#1]:1)~[!#1]~[!#1]~[!#1]~2
6	[!#1]1:[!#1]:[!#1]2:[!#1]:[!#1]:[!#1]:1~[!#1]~2
7	[!#1]1:[!#1]:[!#1]2:[!#1]:[!#1]:[!#1]:1~[!#1]~[!#1]~2
8	[!#1]1:[!#1]:[!#1]2:[!#1]:[!#1]:[!#1]:1~[!#1]~[!#1]~[!#1]~2
9	[!#1]1:[!#1]:[!#1]:[!#1]2:[!#1]:[!#1]:1~2

**10**	[!#1]1:[!#1]:[!#1]2:[!#1]:[!#1]:[!#1]:1~2
**11**	[!#1]1:[!#1]:[!#1]:[!#1]2:[!#1]:[!#1]:1~[!#1]~[!#1]~[!#1]~[!#1]~2
**12**	[!#1]1:[!#1]:[!#1]:[!#1]2:[!#1]:[!#1]:1~[!#1]~[!#1]~[!#1]~[!#1]~[!#1]~2
**13**	[!#1]1:[!#1]:[!#1]2:[!#1]:[!#1]:[!#1]:1~[!#1]~[!#1]~[!#1]~[!#1]~2
**14**	[!#1]1:[!#1]:[!#1]2:[!#1]:[!#1]:[!#1]:1~[!#1]~[!#1]~[!#1]~[!#1]~[!#1]~2
**15**	[!#1&h0]1#[!#1&h0]-[!#1&h0]-1
**16**	[!#1&h0]1#[!#1&h0]-[!#1&h0]-[!#1&h0]-1
**17**	[!#1&h0]1#[!#1&h0]-[!#1&h0]-[!#1&h0]-[!#1&h0]-1
**18**	[!#1&h0]1#[!#1&h0]-[!#1&h0]-[!#1&h0]-[!#1&h0]-[!#1&h0]-1
**19**	[!#1&h0]1#[!#1&h0]-[!#1&h0]-[!#1&h0]-[!#1&h0]-[!#1&h0]-[!#1&h0]-1
**20**	[!#1&h0]1=[!#1&h0]-[!#1&h0]=1
**21**	[!#1&h0]1=[!#1&h0]-[!#1&h0]-[!#1&h0]=1
**22**	[!#1&h0]1=[!#1&h0]-[!#1&h0]-[!#1&h0]-[!#1&h0]=1
**23**	[!#1&h0]1=[!#1&h0]-[!#1&h0]-[!#1&h0]-[!#1&h0]-[!#1&h0]=1
**24**	[!#1&h0]1=[!#1&h0]-[!#1&h0]-[!#1&h0]-[!#1&h0]-[!#1&h0]-[!#1&h0]=1
**25**	[!#1&h0]1=[!#1&h0]-[!#1&h0]-[!#1&h0]-[!#1&h0]-[!#1&h0]-[!#1&h0]-[!#1&h0]=1
**26**	[!#1&h0]1-[!#1&h0]2=[!#1&h0]-[!#1&h0]-1-2
**27**	[!#1&h0]1-[!#1&h0]2-[!#1&h0]-[!#1&h0]=2-1
**28**	[!#1&h0]1=[!#1&h0]2-[!#1&h0]-1-[!#1&h0]-[!#1&h0]-2
**29**	[!#1&h0]1-[!#1&h0]2-[!#1&h0]-1-[!#1&h0]-[!#1&h0]=2
**30**	[!#1&h0]1-[!#1&h0]2=[!#1&h0]-1-[!#1&h0]-[!#1&h0]-2
**31**	[!#1&h0]1=[!#1&h0]2-[!#1&h0]-1-[!#1&h0]-[!#1&h0]-[!#1&h0]-2
**32**	[!#1&h0]1-[!#1&h0]2-[!#1&h0]-1-[!#1&h0]-[!#1&h0]-[!#1&h0]=2
**33**	[!#1&h0]1-[!#1&h0]2=[!#1&h0]-1-[!#1&h0]-[!#1&h0]-[!#1&h0]-2
**34**	[!#1&h0]1=[!#1&h0]2-[!#1&h0]-1-[!#1&h0]-[!#1&h0]-[!#1&h0]-[!#1&h0]-2
**35**	[!#1&h0]1-[!#1&h0]2-[!#1&h0]-1-[!#1&h0]-[!#1&h0]-[!#1&h0]-[!#1&h0]=2
**36**	[!#1&h0]1-[!#1&h0]-[!#1&h0]2-[!#1&h0]=1-[!#1&h0]-[!#1&h0]-2

**37**	[!#1&h0]1-[!#1&h0]-[!#1&h0]2-[!#1&h0]=1-[!#1&h0]-[!#1&h0]-[!#1&h0]-2
**38**	[!#1&h0]1-[!#1&h0]-[!#1&h0]2-[!#1&h0]-1=[!#1&h0]-[!#1&h0]-[!#1&h0]-2
**39**	[!#1&h0]12-[!#1&h0]-[!#1&h0](-[!#1&h0]-1)-[!#1&h0]=2
**40**	[!#1&h0]12-[!#1&h0]-[!#1&h0](-[!#1&h0]=1)-[!#1&h0]-[!#1&h0]-2
**41**	[!#1&h0]12-[!#1&h0]-[!#1&h0](-[!#1&h0]-1)-[!#1&h0]-[!#1&h0]=2
**42**	[!#1&h0]12-[!#1&h0]-[!#1&h0](-[!#1&h0]=1)-[!#1&h0]-[!#1&h0]-[!#1&h0]-2
**43**	[!#1&h0]12-[!#1&h0]-[!#1&h0](-[!#1&h0]-1)-[!#1&h0]-[!#1&h0]-[!#1&h0]=2
**44**	[!#1&h0]12=[!#1&h0]-[!#1&h0](-[!#1&h0]-[!#1&h0]-1)-[!#1&h0]-[!#1&h0]-2
**45**	[!#1&h0]12-[!#1&h0]-[!#1&h0](-[!#1&h0]-[!#1&h0]-1)-[!#1&h0]-[!#1&h0]=2
**46**	[!#1&h0]12-[!#1&h0]-[!#1&h0]-[!#1&h0](-[!#1&h0]-[!#1&h0]-[!#1&h0]-1)-[!#1&h0]=2
**47**	[!#1&h0]12=[!#1&h0]-[!#1&h0]-[!#1&h0](-[!#1&h0]-[!#1&h0]-[!#1&h0]-1)-[!#1&h0]-2
**48**	[!#1&h0]12-[!#1&h0]-[!#1&h0]-[!#1&h0](-[!#1&h0]-[!#1&h0]-[!#1&h0]=1)-[!#1&h0]-2
**49**	[!#1&h0]12-[!#1&h0]-[!#1&h0]-[!#1&h0](-[!#1&h0]-[!#1&h0]=1)-[!#1&h0]-[!#1&h0]-2
**50**	[!#1&h0]123-[!#1&h0](-[!#1&h0]-1-[!#1&h0]-2)-[!#1&h0]-3

5.2 Conformer Generation and Geometry Optimization

Molecule conformers were generated with the *ETKDGv3* algorithm[6] in RDKit. The number of conformers per molecule was set to 10. All successfully generated conformers were then optimized with the Universal Force-Field (UFF)[5]. The maximum number of iterations was set to 500. Conformers, for which the geometry optimization did not converge were discarded. Only molecules for which at least one conformer was successfully optimized, moved on to the next stage.

5.3 Distorted Angle and Bond Filter

While the geometry optimization with the UFF force field has the advantage of substantially faster than more sophisticated methods, the optimized structures tend to be less accurate[7]. To this end, bond

and angle parameters were compared against reported structures to filter out potentially invalid molecules.

5.3.1 Database construction of bond and angle-parameter distributions

The 3d structures of 613290 molecules from the CCDC database containing only elements C, H, N, O, P, S, Cl, Br, F, I, B, and Si were extracted to compute bond and angle distributions of bond and angle types. All bond lengths and angles were extracted and stored in a relational database, resulting in a total of 138110588 angles and 37993129 bond parameters.

5.3.2 Gaussian Mixture Models of Bond and Angle Parameters

The geometry optimizations of generated molecule conformers were performed with a time-efficient, but crude approximation via a force field optimization. To validate that the generated geometries represent realistic molecules, the bond and angle parameters of each conformer were compared against reported distributions of the respective bond or angle type. Gaussian Mixture Models (GMMs) were used to approximate the distribution of literature reported (from the CCDC database) values found for a given bond or angle type. To do so, bonds were grouped by atom of element type A and element type B connected by a given bond valence (single, double, triple or an aromatic bond). Angles were grouped equally. The number of Gaussian (components) to be used for the GMM was determined via the Bayes Information Criterion (BIC). The maximum number of components was set to 10 with a maximum number of optimization iterations per GMM of 3000. The approximation of the distributions with a GMM was performed with scikit-learn[8]. The number of components was set to n for which $n+1$ reduced less than a threshold values of 0.01 as measured by the BIC (Supplementary figs. 8 and 9). The GMM were calculated for all parameters for which at least 500 examples had been extracted from the CCDC database as detailed above (section 5.3.1). For parameters with fewer examples, no GMMs were calculated.

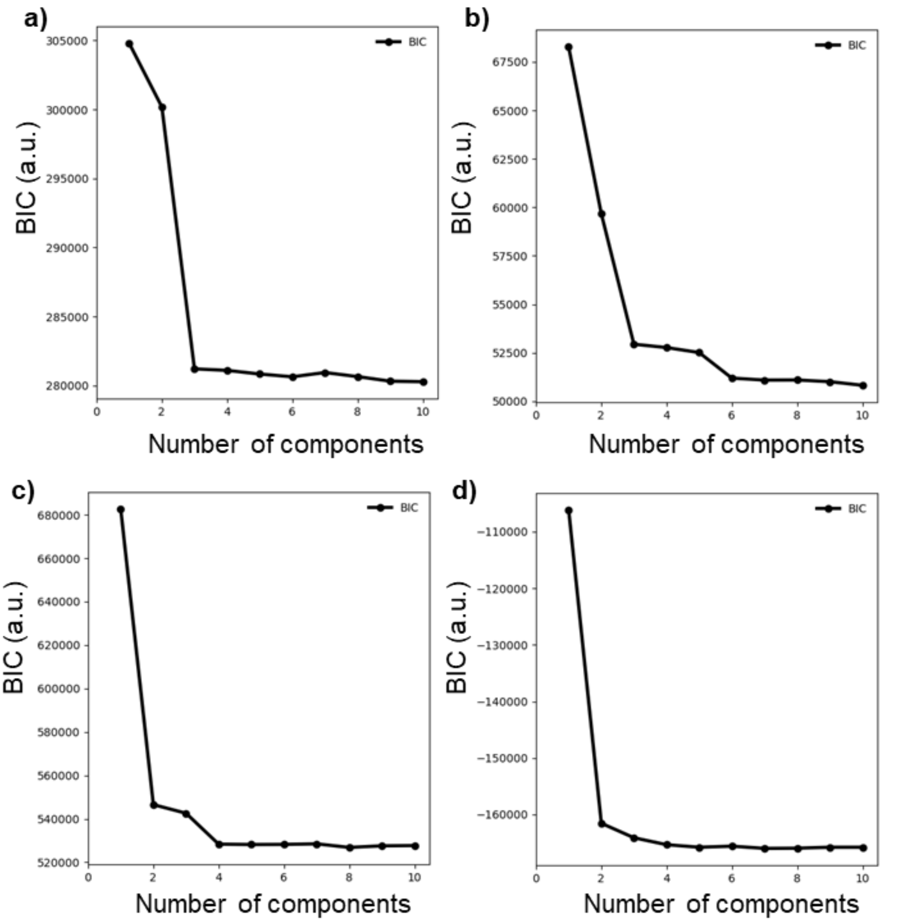

Supplementary figure 8: Examples of determining the number of Gaussian distributions in a GMM for approximating the distribution of a bond or angle type. The number of gaussian distributions (number of components) to use for fitting a GMMs to the parameter distributions was determined by the BIC. Examples of the BIC are shown for the angle, or bond distributions of C-C-C, C-B-O , Cl-P-N, and C#C (**a** to **d**).

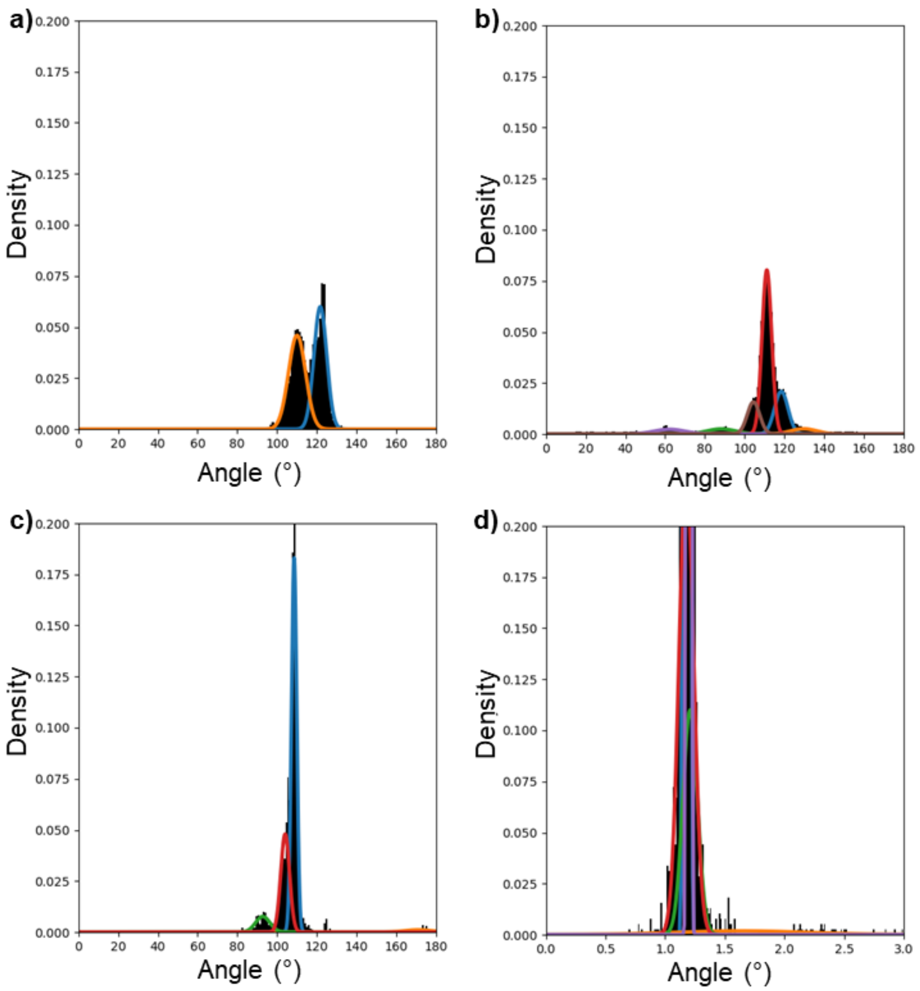

Supplementary figure 9: Examples of GMMs fitted to angle distributions. a-d) GMMs were fitted to the angle distributions of C-B-O, C-C-C, Cl-P-N, and C#C with the number of Gaussian distributions as defined above.

5.3.4 Filtering of unusual bond- and angle parameters

The bond and angle parameters of molecules for which at least one conformer was successfully generated, and geometry optimized, were compared against the parameter distributions in reported structures approximated with GMM as described in section 5.3.3. A given bond or angle of a conformer passes this stage if it lies within four standard deviations of any of the Gaussian distributions of a GMM. Since the CCDC database only contains so far discovered (though a good estimation) of plausible bond and angle parameter values, a molecule conformer was allowed to fail this test on one parameter for every 5 atoms in the molecule, as to not disallow all parameters not within observed parameter distributions. For a molecule to pass this stage, at least one of its conformers had to pass

this stage. A precalculated set of GMM distributions is provided within the code for all relevant bond lengths and angle types.

5.4 Disallowed Substructure Filter Examples

The *disallowed substructure filter* as described was tested on 10000 randomly sampled molecules from the coconut database. Out of these, 7 (0.07%) did not pass this filtering stage (compare Supplementary fig. 10).

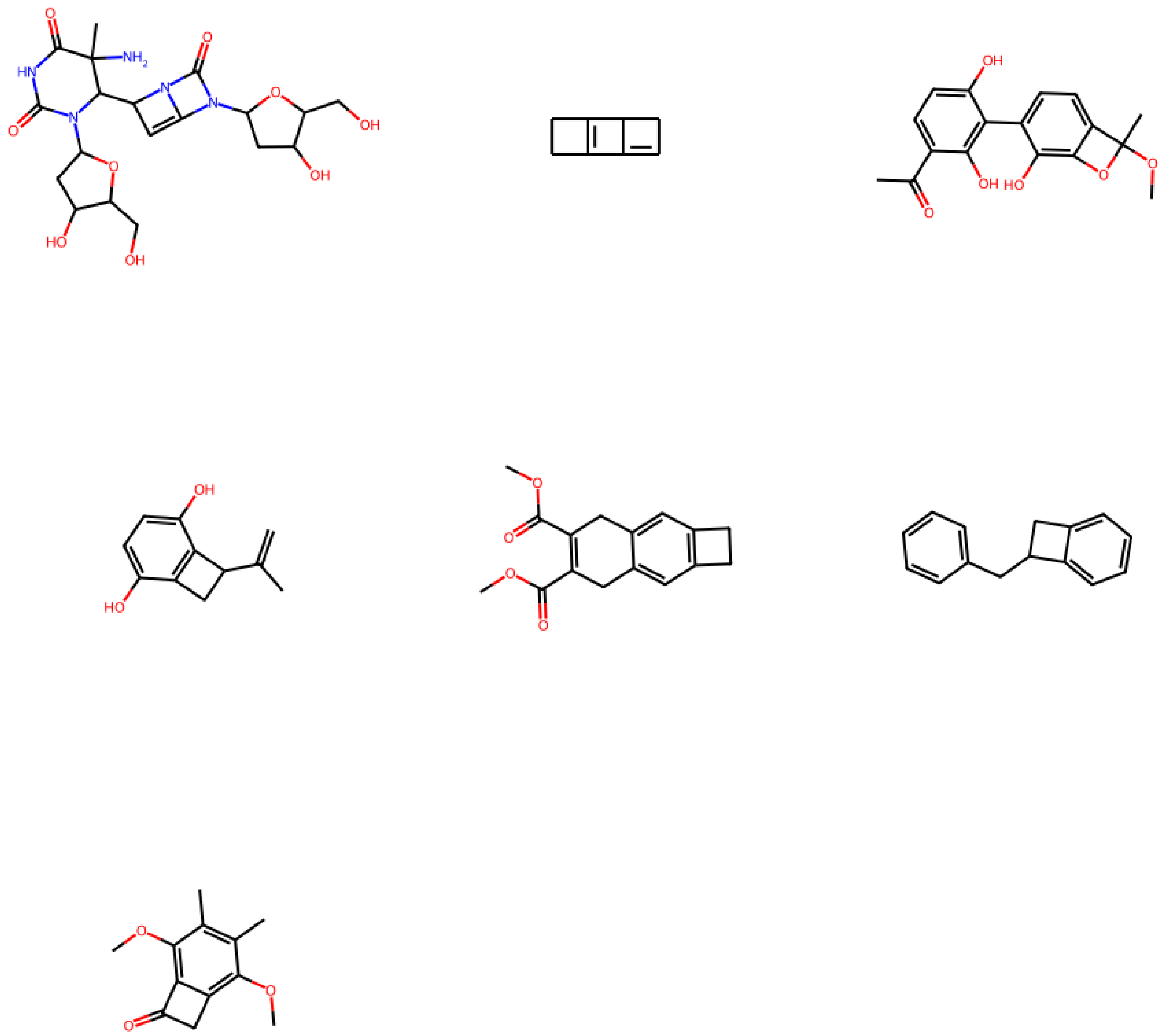

Supplementary figure 10: Examples of molecules that contained disallowed substructures. 7 out of 10000 randomly sampled molecules from the COCONUT database did have at least one disallowed substructure.

5.5 Conformer and Geometry Optimization Examples

Equally, the conformer and Geometry Optimization were tested on 10000 randomly sampled molecules from the COCONUT database with a maximum of 50 bonds. Out of those, 26 did not result in an energy-minimized conformation. The number of conformers and hyperparameter settings were set as described above.

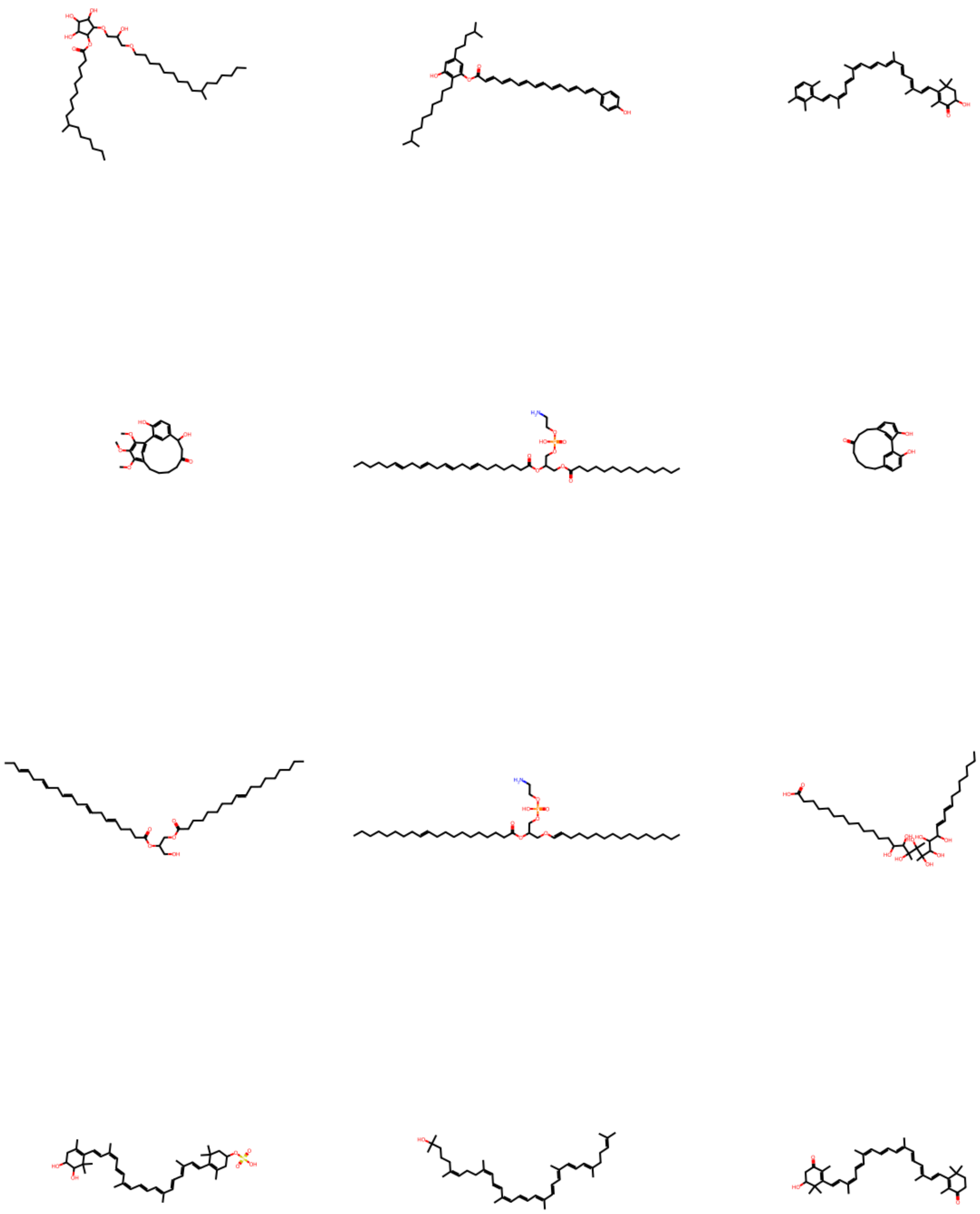

Supplementary figure 11: Examples of molecules for which either no conformers could be generated, or failed to be geometry optimized. 12 out of the 26 molecules for which no conformers could be generated or none of the generated conformers was successfully geometry optimized.

5.6 Distorted Bond and Angle Filter Examples

To test the bond and angle filter by comparing with reported structures, 10000 randomly sampled molecules from the COCONUT database with a maximum of 50 bonds were tested. Since conformers have to be generated for these first, only those molecules that passed the conformer generation and energy minimization stage were considered. Out of the 10000 molecules, 23 did not pass the conformer generation and energy minimization stage. From the remaining 9977 molecules, 1625 molecules, failed this filtering stage. A selection of these molecules is presented in Supplementary fig. 12.

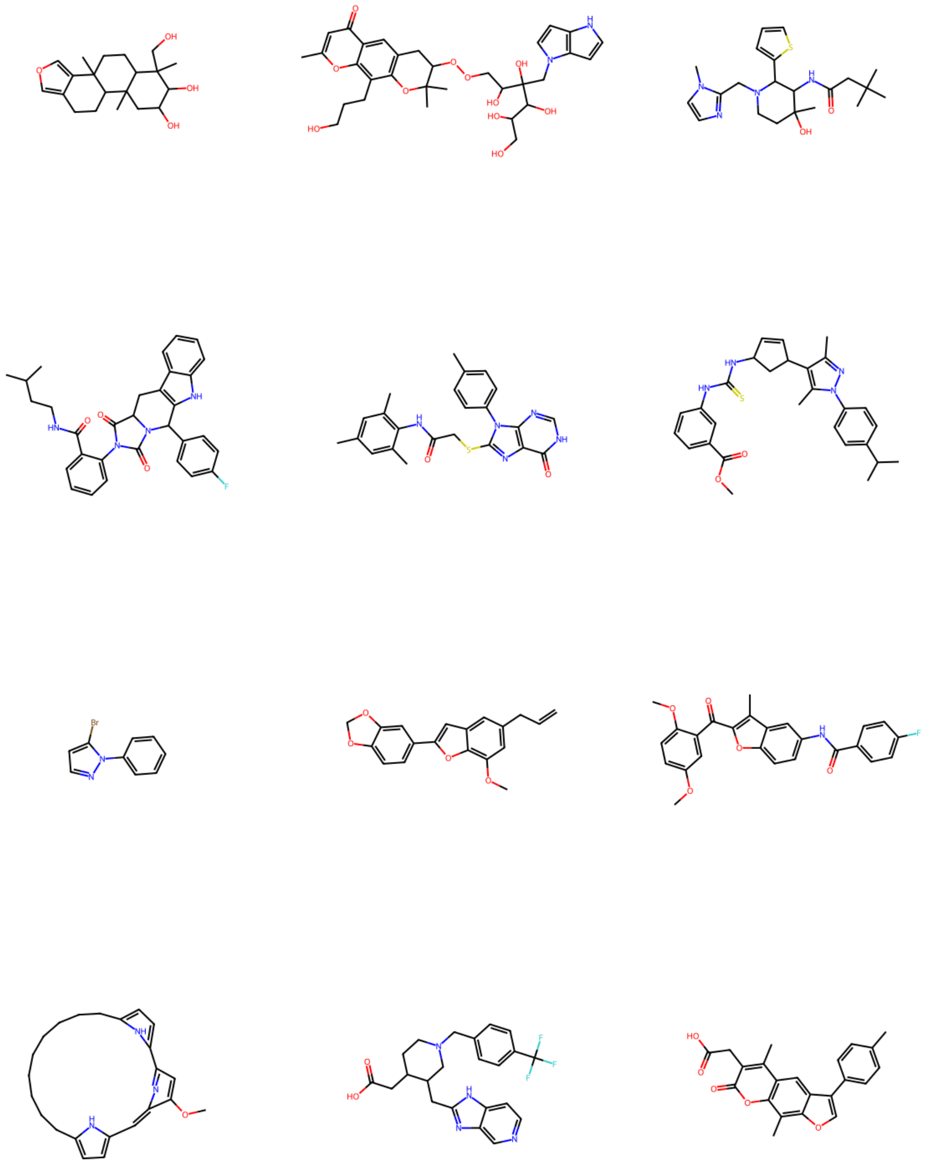

Supplementary figure 12: Example of molecules for which generated conformers contained too many distorted bonds or angles. 12 out of the 1625 molecules that did not pass the distorted bond and angle parameter stage.

6 Divergence estimation

6.1 Molecular Similarity Quantification

Molecular fingerprints are typically used to quantify the similarities between two or a set of molecules. Here we calculated the Morgan Fingerprints[3] (radius=2, bits=512) as a basis for molecular similarity estimations. To quantify the similarity between two molecules the Dice-Similarity[9] (Equation 1) was calculated which is an extension of the Jaccard Index.

$$s = \frac{2|a \cdot b|}{|a|^2 + |b|^2} \tag{1}$$

where s is the similarity, between binary vectors a and b.

6.2 JAS Similarity Quantification

To quantify the similarity between two JASs, first all fingerprints for building blocks, contingent-, and observed-objects were calculated. The similarity was then calculated by taking the pairwise similarity between all objects from a given assembly depth d between the two JAS. The similarity was calculated by taking the average highest similarity between each molecule from the first JAS in an assembly depth, and all objects from the same assembly depth in the second JAS. The divergence of a JAS relative to another was calculated by $1 - s$ for each assembly depth in the two JAS, where s is the similarity defined by Equation 1.

7 Influence of Molecule Generation Hyperparameters

7.1. Influence of Adjusting Sampling Weights for Assembly Depth Selection

To assess the influence of adjusting the sampling weights for selecting the assembly depth from which to sample a molecule, the JAS of 10,000 randomly sampled molecules from the COCONUT database was created. A contingency loss down to assembly depth 10 was induced and JAS subsequently

regenerated. The sampling weights for the layer selection were then adjusted by scaling c (Equation 2) for each time the JAS was regenerated.

$$p_{di} = \sum_{i=0}^{n} f_i^{\ c} \quad (2)$$

where f_i is the sum of the number of pathways that all fragments from assembly depth i were employed in. c is a user-selected parameter to scale the selection of the assembly depth to sample from. Intuitively $c = 0$, results in an equal probability of an assembly depth being selected independent of the fragments, and $c = 1$ scales the probability of selection proportional to the number of pathways the given fragments of an assembly depth have been employed in. Scaling c naturally results in shifting distributions of the resulting molecules as displayed in Supplementary fig. 13.

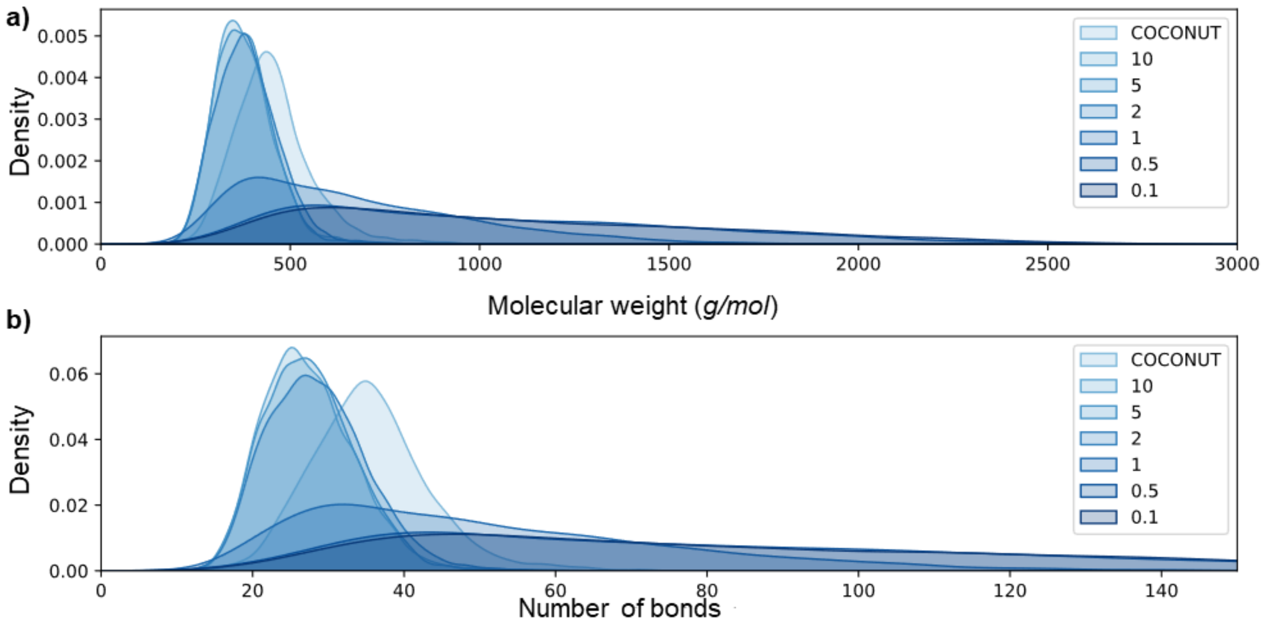

Supplementary figure 13: Influence of altering the assembly depth selection on molecular weight and number of bonds of reconstructed molecules. Molecular weight (**a**)) and number of bond (**b**)) distributions of 10000 generated molecules using the algorithm described above. The molecules were generated from the JAS of 10000 randomly selected natural products from the COCONUT database with a maximal assembly depth of 20. From this JAS 10000 new molecules were generated after removing contingency by assembly depth (see above) down to assembly depth 10. For regeneration of the JAS, the scaling factor c (Equation 2) was scaled as indicated in the figure.

7.2 Influence of Adjusting Sampling Weights for Fragment Selection

Similar to selecting the sampling weight for selecting the assembly depth to sample an object from, the sampling weights for selecting a fragment having selected an assembly depth to sample from were set by equation 3.

$$p_{fi} = \frac{f_{i,outdegree}^{s}}{\sum_{i}^{n} f_{i,outdegree}^{s}} \tag{3}$$

Given a selected assembly depth, the probability of selecting a fragment f_i is set to be the relative number of outgoing edges in the JAS of this fragment compared to all fragments within this JAS, with the same assembly depth. Optionally this probability (i.e. sampling weight) was scaled by the exponent s. Intuitively, setting $s = 0$, results in a uniform probability of selecting fragments from an assembly depth, whereas $s = 1$ scales the probability proportional to the number of outgoing edges. To test the influence of changing s the JAS of 10000 randomly sampled natural products from the COCONUT database was generated. Contingency of this JAS was then removed down to assembly depth 10. The JAS was subsequently regenerated by scaling the exponential factor s. Changing this exponent s results in drastically changing molecule distributions, as highlighted in Supplementary fig. 14.

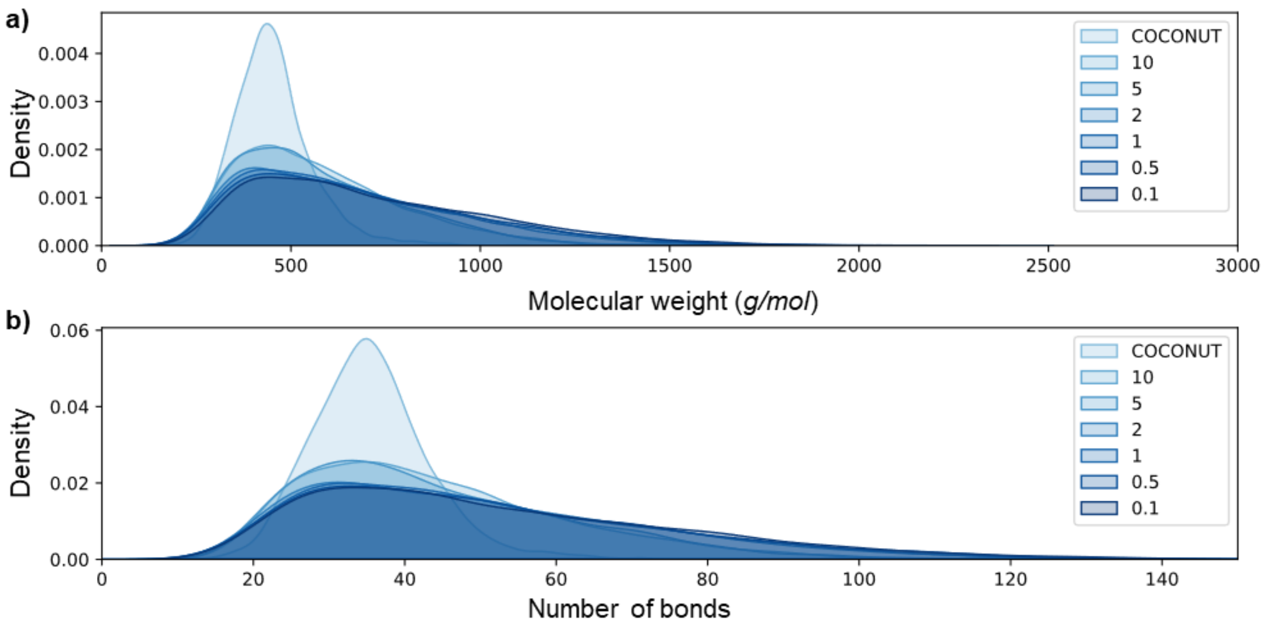

Supplementary figure 14: Influence of altering the fragment selection on molecular weight and number of bonds of reconstructed molecules. Adjusting the sampling weights for selecting a fragment at a specific assembly depth given a JAS alters the distribution of molecular weight (**a)**) and number of bonds (**b)**) of generated molecules. To test the influence, the JAS of 10000 randomly sampled natural products from the COCONUT database with a maximal assembly depth of 20 was constructed. From this JAS 10000 new molecules were constructed, after removing contingency by assembly depth (see above) down to assembly depth 10. For regenerating the JAS, the scaling factor *s* was adjusted as indicated in the figure.

7.3 Influence of adjusting sampling weights for the number of construction steps selection

Since the observed objects in a JAS can take varying numbers of construction steps to be assembled, matching the distribution of the number of construction steps of the original JAS, when reconstructing JAS, is critical to match the chemical distributions. For each newly generated molecule, the number of construction steps is sampled according to where the probability of selecting a number of construction steps is set according to equation 4.

$$p_n = \frac{[\sum_{d=n\,;i} 1]^l}{\sum_d [\sum_i \ 1]^l} \tag{4}$$

where p_n is the probability of sampling n number of construction steps to construct a molecule starting from a given assembly depth (i.e. if construction starts from assembly depth 10 and the maximal

assembly depth is 20 n is in the range [1, 10]). d is the assembly depth and in the range of the lowest assembly depth of an observed object in a given JAS to the highest assembly depth of an observed object in the JAS. In the case that the reconstruction of the JAS starts from an assembly depth larger than the minimal assembly depth of an observed object in the original JAS, the lower bound for the number of steps is adjusted accordingly. Similarly to the scaling of the sampling weights for the assembly depth and fragment selection, the sampling weights for selecting the number of construction steps were optionally scaled by the exponential scaling factor l. Intuitively, the probability of sampling n number of construction steps is proportional to the number of observed objects in an assembly depth divided by the total number of observed objects in the JAS, starting from the assembly depth at which the construction of new molecules begins +1. Setting the exponential scaling factor $l = 0$, results in a uniform probability of a number of construction steps to be taken, whereas $l = 1$ scales the probability proportional to the number of observed objects in the original JAS. The influence of scaling l on the distribution of molecular weight and number of bonds of observed objects in a reconstructed JAS is shown in Supplementary fig. 15. The JAS of 10000 randomly selected natural products from the COCONUT database with a maximal assembly depth of 20 was constructed, and contingency was removed down to assembly depth 10. To reconstruct the JAS, the exponential factor l was then scaled as indicated the Supplementary fig. 15.

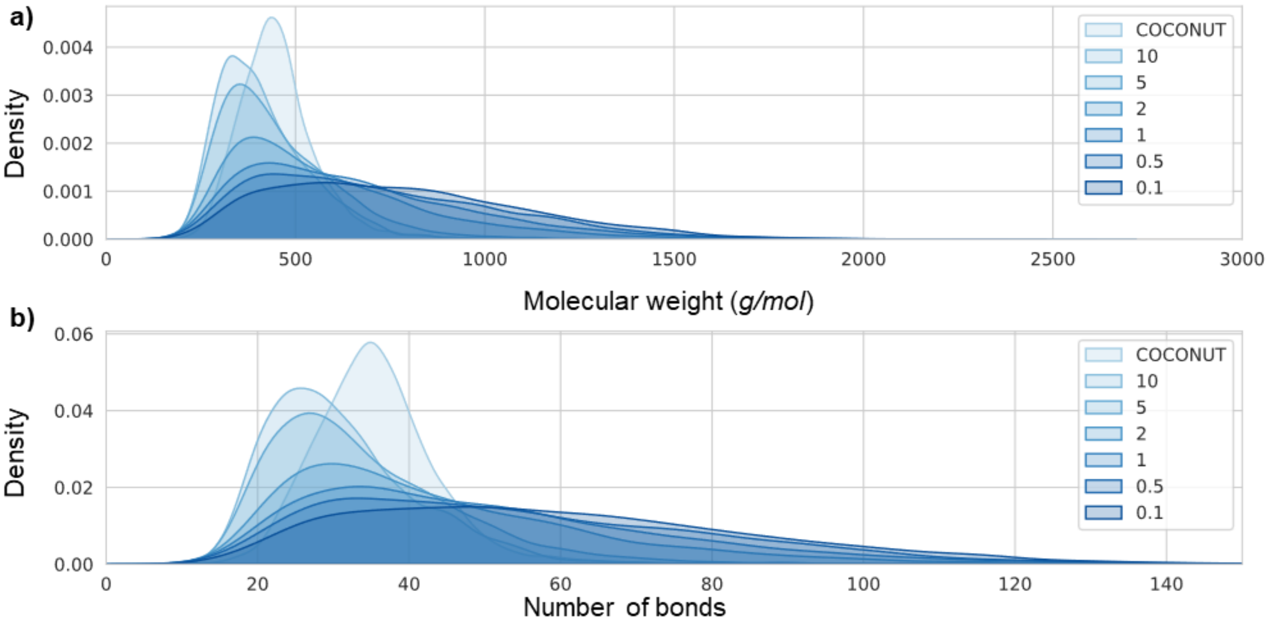

Supplementary figure 15: Influence of altering the number of construction steps selection on molecular weight and number of bonds of reconstructed molecules. Adjusting the sampling weights for selecting the number of construction steps to be taken as per equation 4 results in shifting distributions in molecular weight (**a)**) and number of bonds (**b)**) in constructed molecules. The JAS of 10000 randomly sampled natural products from the COCONUT database with a maximal assembly depth of 20 was constructed. The JAS was reconstructed, after removing contingency by assembly depth (see above) down to assembly depth 10. For regenerating the JAS, the scaling factor l was adjusted as indicated in the figure.

8 Reconstruction of molecules from partial Contingency

In the following section details for the reconstruction of molecules from the contingency of the natural products to estimate the divergence of JAS with optional selection pressure, as well as the construction of drug-like molecules are provided. Additionally, details are provided for the reconstruction of molecules from the assembly pathways of Brefelamide.

8.1 Reconstruction of molecules for the estimation of divergence

From the JAS of natural products, contingency was removed ($\omega = \{2, 6, 10, 14\}$) by removing objects (observed objects as well as objects on their pathways), if these objects had an assembly depth larger or equal to $20 - \omega$ (20 was the maximal assembly depth in the JAS). For the reconstruction of molecules that mimic the JAS of natural products the sampling weights were set to $l = 1, s = 1, c = 2$. The

reconstruction of the molecule to diverge from the JAS of natural products was performed with sampling weights set to $=1, s=0, c=2$.

8.2 Reconstruction of molecules for the estimation of the exploration rates

For the reconstruction of molecules with alternate selection pressure ($s=\{0.5, 1, 5\}$) contingency $\omega = 23$ was removed from the JAS of natural product of molecules with an assembly depth of up to 25. All objects with an assembly depth larger than $25-\omega$ were removed. Molecules were then reconstructed from the remaining objects. The sampling weights were set to $l=1, s=1, c=2$.

For the reconstruction of molecules after differing contingency loss $\omega=\{17, 19, 21\}$ observed objects, as well as all sub-objects only existing on removed objects pathways, were removed. Molecules were reconstructed from the remaining objects with the same sampling weight settings as described above.

8.3 Reconstruction of molecules for the generation of drug-like molecules

The reconstruction of drug-like molecules from the JAS of natural products was performed after removing contingency down to assembly depth 5 ($\omega=15$). 10000 molecules were constructed as described above. The sampling weights were set to $l=1, s=1, c=2$. Additionally to the above-described filters, PAINS substructure filters[10] were used to filter out molecules commonly blacklisted in drug discovery campaigns. Furthermore, the number of atoms by which two fragments are combined was adjusted to mirror the observed distributions in the JAS of drug-molecules modelled as described above.

8.4 Reconstruction of molecules from the assembly pathway of Brefelamide

10000 molecules were reconstructed from the assembly pathway of Brefelamide after removing contingency ($\omega=\{0, 1, \ldots, 10\}$). The sampling weights for the reconstruction of molecules were set to $l=1, s=1, c=2$. Molecules were filtered using the filtering pipeline described above.

9 Database Analysis

The Assembly Possible and Assembly Observed spaces were modelled using the chemical databases of PubChem[11] and COCONUT[12], respectively. In the following sections, some of the relevant properties of these databases for this work were analysed.

9.1 Number of Bonds Distribution in PubChem and COCONUT Databases

While the PubChem database serves as an approximation of the complete representation of molecules on a planetary scale, the COCONUT database is an approximation of the organic molecules with biological origin. This includes all primary and secondary metabolites and is considered to be the chemical space as an outcome of evolutionary processes on planetary scale. A rough first estimation of the MA of a molecule can be made upon the number of bonds present in that given since the upper- and lower-bounds of the possible MAs are n and $log_2(n)$ respectively, where n is the number of bonds. The distribution of the number of bonds of the molecules in the PubChem, and COCONUT database shows a shift to molecules with a larger number of bonds in the molecules of the COCONUT database (Supplementary fig. 16).

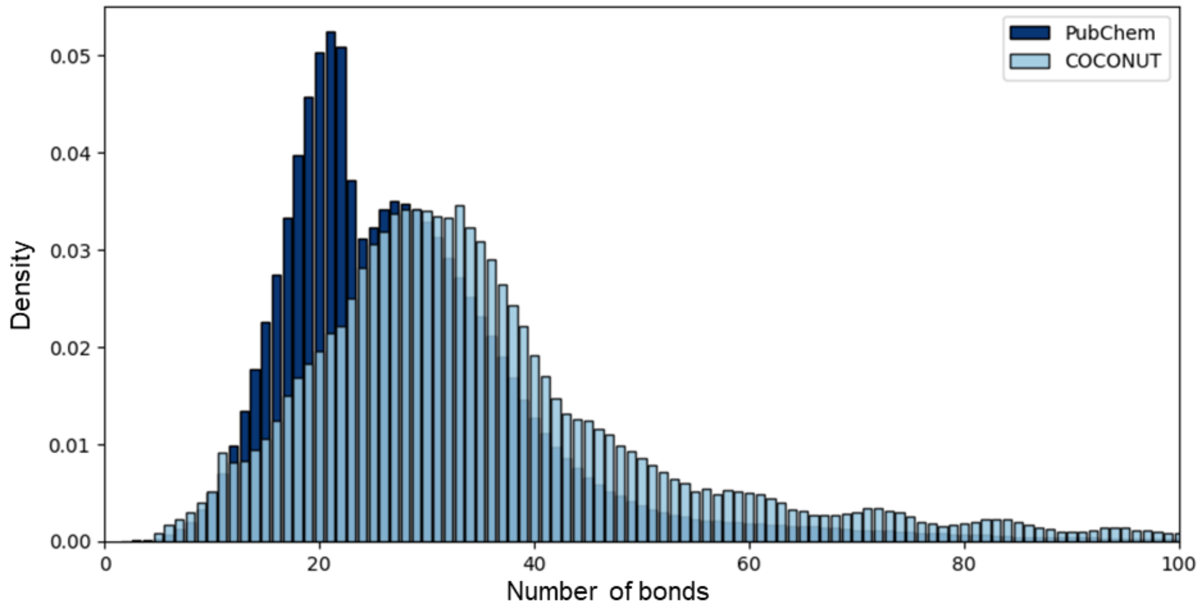

Supplementary figure 16: Kernel Density Estimate (Density) of the number of bonds of all molecules in the COCONUT and PubChem databases.

9.2 Object Distributions in PubChem and COCONUT Databases

The relative number of objects (building blocks, contingent-, and observed objects) in the assembly spaces of all molecules in the COCONUT (with a maximal assembly depth of 25), or PubChem databases was analysed after calculating all the assembly pathways of the molecules in either database as described above. As shown in Supplementary fig. 17, there is a shift in the maximum of the maximal number of objects per assembly depth between the COCONUT and PubChem databases. While the maximum in the case of the PubChem database is at assembly depth 9, the number of unique objects is greatest at assembly depth 8 in the COCONUT assembly pathways. In contrast to the maximum number of objects, the distribution of unique objects in the case of the COCONUT database, has relatively more unique objects at larger assembly depths, compared to the PubChem database.

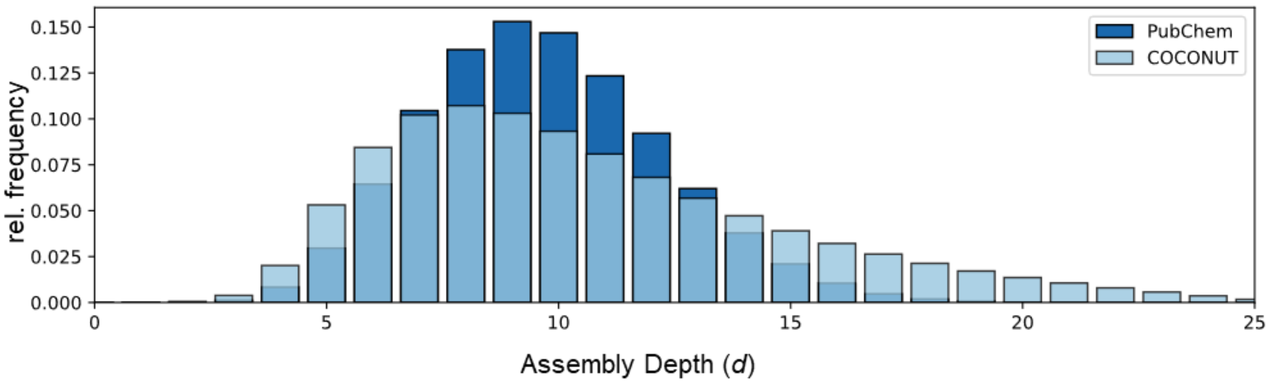

Supplementary figure 17: Distribution of the relative number of unique objects per assembly depth compared to the number of unique objects among all assembly pathways within the COCONUT and PubChem databases respectively. Only the molecules of which the assembly pathways, as described above, were calculated were considered for this analysis.

Consequently, the exploration of the unique objects in the assembly space of PubChem occurs at lower assembly depths compared to the assembly space of the molecules of the COCONUT database (Supplementary fig. 18). While the exploration rate (accumulative number of unique objects explored up to a certain assembly depth relative to the total number of unique objects) reaches almost full

exploration at assembly depth 15 in the assembly space of PubChem molecules, more objects are still being explored in the assembly space of the COCONUT molecules.

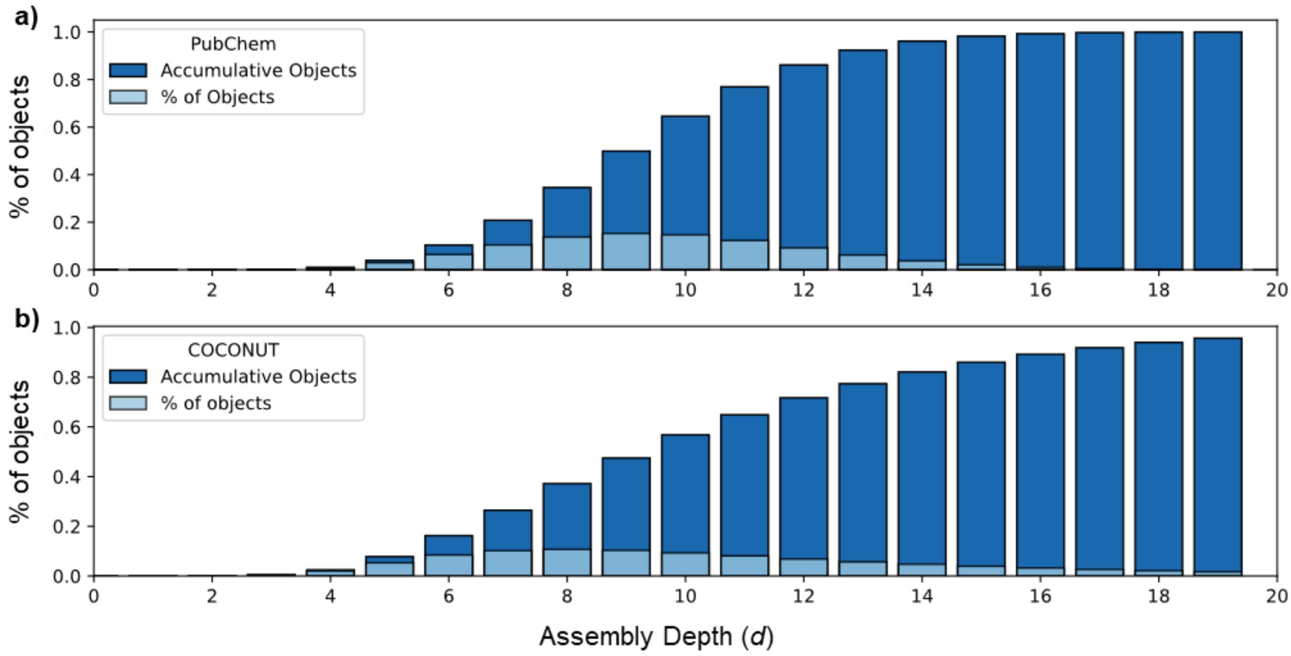

Supplementary figure 18: Accumulative, relative number of unique objects, as well as relative number of unique objects per assembly depth. All calculated assembly pathways of the molecules in the PubChem (**a)**), and COCONUT database (**b)**) were considered for this analysis.

The difference in exploration rates can be clearly explained by the percentage of unique contingent (objects on observed objects pathways) vs. observed objects (objects, in our case molecules, that are physically observed i.e. present in the respective databases) per assembly depth relative to the total number of unique objects in an assembly space. The assembly space of COCONUT molecules has a higher amount of unique contingent, as well as observed objects at higher assembly depth relative to the PubChem assembly space (Supplementary fig. 19). Additionally, the ratio of unique contingent to unique observed objects is notably higher in the COCONUT than in the PubChem assembly space.

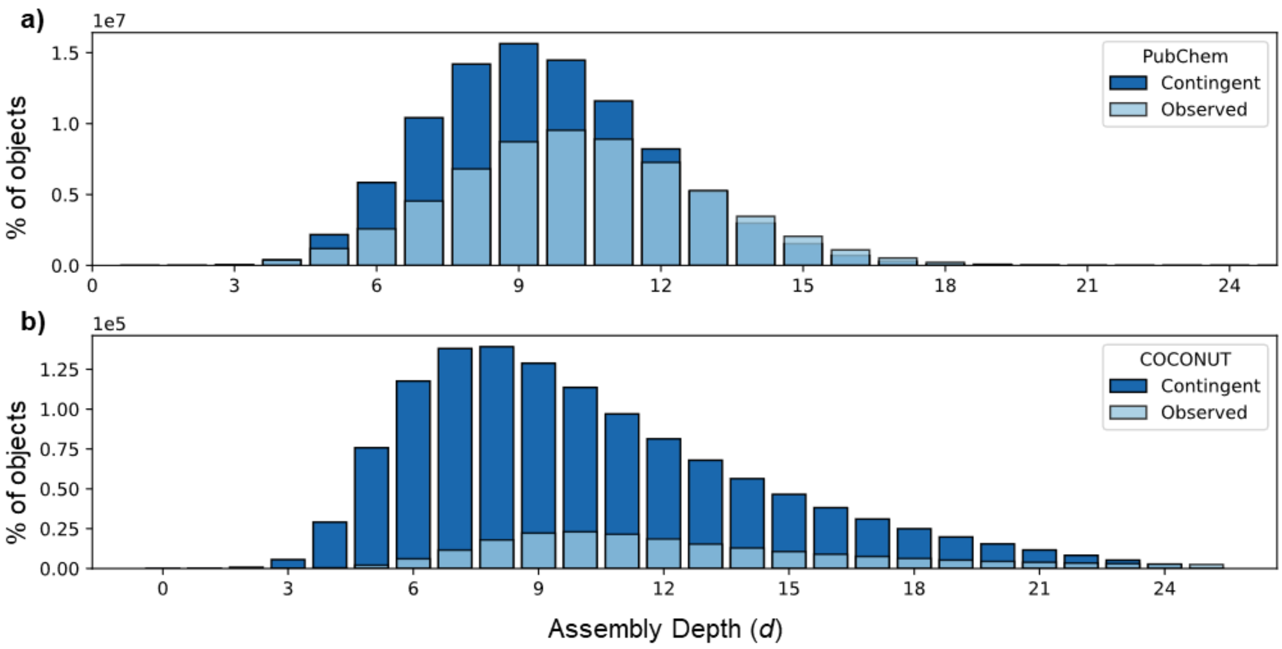

Supplementary figure 19: Distribution of contingent and observed objects in the assembly pathways of the molecules of the PubChem, and COCONUT database. All assembly pathways of the molecules in the PubChem (**a**)), and COCONUT database (**b**)) were considered for this analysis.

9.3 Object Utilization in the JAS of COCONUT and PubChem

To analyse the utilization of objects in the JAS of COCONUT or PubChem molecules, the JAS were constructed as described above. In the case of the PubChem JAS, the JAS was constructed from 500k randomly sampled molecules of the molecules that the assembly pathway was calculated for. As shown in Supplementary fig. 20, objects of higher assembly depth in the JAS of the COCONUT database were utilized to construct a new object, compared to objects in the JAS of PubChem molecules, indicating stronger contingency on shared fragments in the JAS of COCONUT (Assembly Observed).

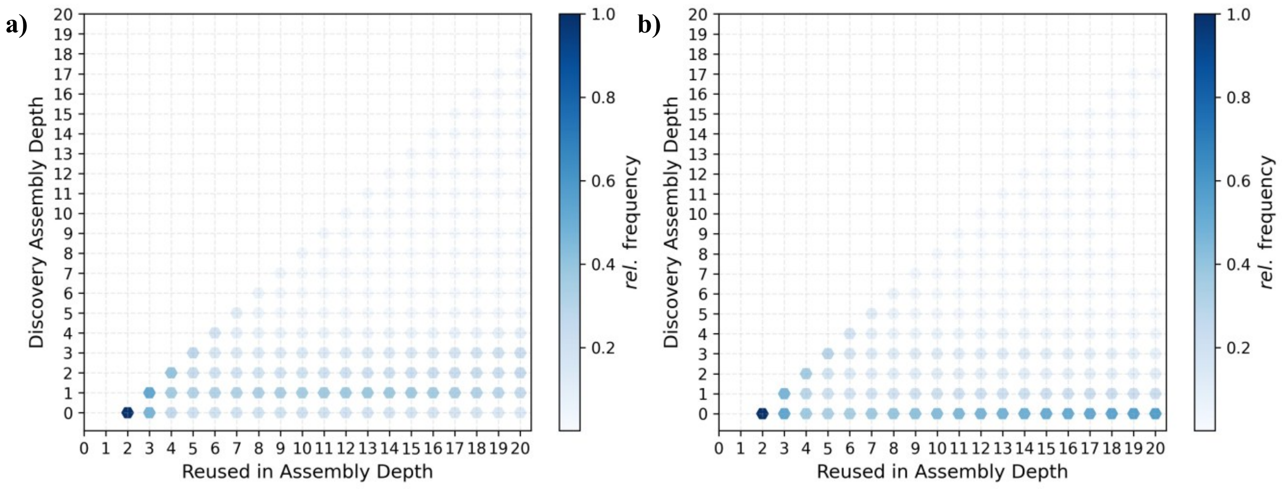

Supplementary figure 20: Frequency of discovered objects being utilized for construction of objects at subsequent assembly depths. The y-axis represents the assembly depth at which objects have been discovered (i.e. building blocks are at assembly depth 0, and objects that have been constructed from two building blocks at assembly depth 1). The x-axis, in contrast, represents the assembly depth at which discovered objects have been used to construct new objects. The intensity of the respective point indicates how frequently an object from a certain assembly depth has been used to construct an object from that assembly depth. The frequencies were normalized per value of the x-axis "Reused in Assembly Depth". **a)** COCONUT JAS; **b)** PubChem JAS.

10 Quantification of exploration in Joint Assembly Spaces

To quantify the exploration of a parent JAS by a JAS subspace, the ratio of total objects per assembly depth was calculated and approximated with function 5.

$$\frac{N_X}{N_P} = k \cdot e^{-\beta d} \tag{5}$$

$\frac{N_X}{N_P}$ represents the exploration rate, d the assembly depth, β is a characteristic constant and the pre-exponential factor k will be the ratio of building blocks when comparing two JASs. The optimal parameter β for two given JASs was calculated using the Python module *scipy*[13] (1.12.0). The pre-exponential factor m was fixed for any given JASs to the ratio of building blocks.

10.1 Exploration rate of JAS_{PC} by JAS_{C} considering only COCONUT building blocks

For the calculation of the exploration rate of the JAS of PubChem molecules (JAS_{PC}) only containing objects with building blocks present in the JAS of COCONUT molecules (JAS_{C}) first all objects containing any building block not in JAS_{C} (see Supplementary table 3 for JAS_{C} building blocks) were removed from JAS_{PC}. The exploration rate was estimated as described above yielding a characteristic constant $\beta = 1.299$.

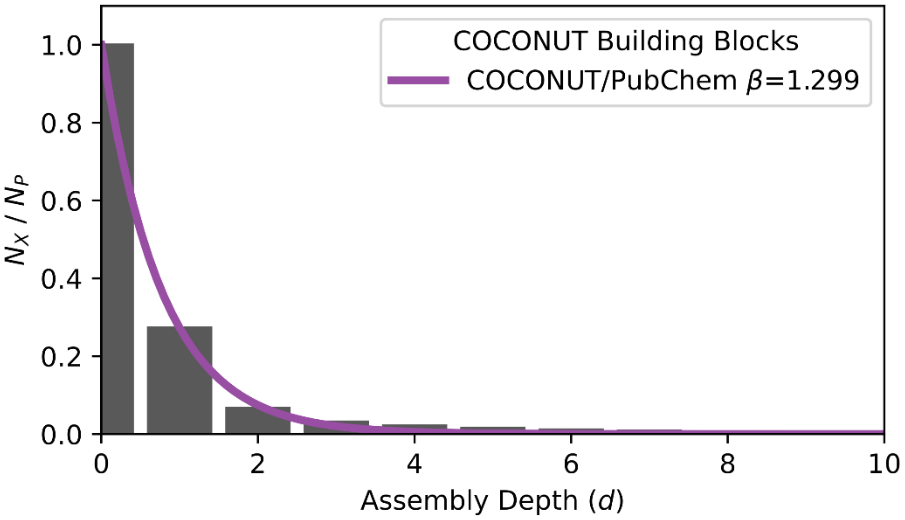

Supplementary figure 21: Exploration rate of JAS_{PC} by JAS_{C} considering only fragments containing building blocks found in JAS_{C}. The grey bars represent the ratio of total objects per assembly depth between JAS_{C} and JAS_{PC}. The purple line present the approximation of the exploration rate by equation 5.

Supplementary table 3: Smiles representation of the bond types found in JAS_{C}

C=O	CCl	NBr	O=Se
CO	OO	BN	C[SeH]
CC	C#N	BO	O=[IH]
CN	C#C	PS	[SeH][SeH]
C=C	OP	P=S	SCl
C=S	O=P	N#N	N=P
NN	CI	NI	[SiH3][SiH3]

C=N	SS	BC	NCl
NS	N=N	S=S	FP
O=S	O[SiH3]	PP	BCl
CS	C[SiH3]	N=S	N[SeH]
CBr	CP	N[SiH3]	S[SeH]
CF	[SiH3]Cl	C=P	OI
NO	N=O	FS	OCl
OS	NP	O[SeH]	

11. Analysis of lost molecular structures

Molecular structure may be gained and lost during the evolutionary process as shown by Klapper, M. *et al.*[14]. This can lead entire metabolites disappearing from the pool of natural products. Although the exact metabolite structure may be lost and therefore not perfectly captured by the JAS of natural products presented in this work, many substructures and close analogues are preserved (see Figure S22 for comparison).

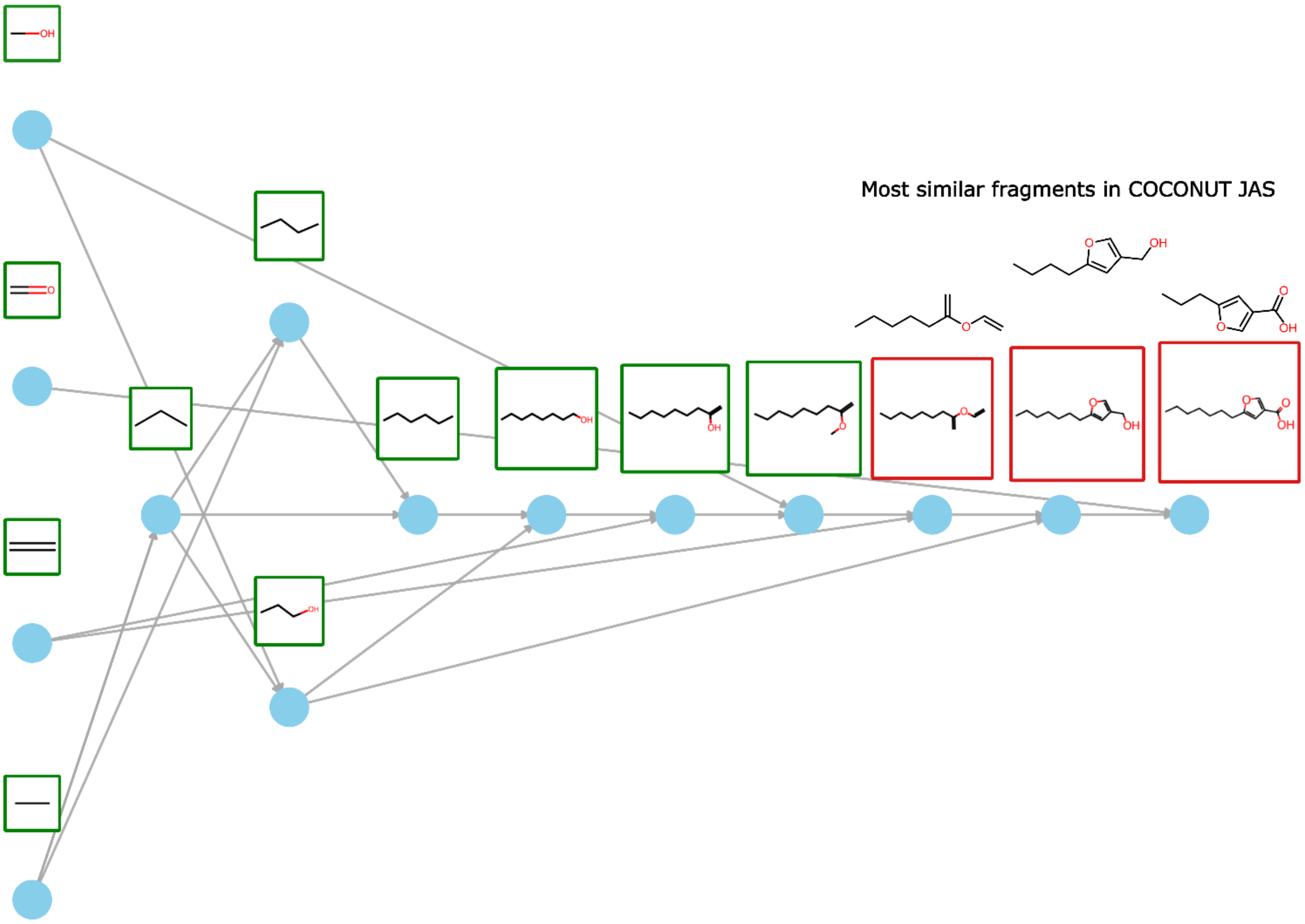

Supplementary Figure 22: Analysis of the assembly fragments of a *lost* metabolite from an ancient biosynthetic gene clusters[14]. The assembly pathway of 5-heptylfuran-3-carboxylic acid is shown. Fragment exactly contained in the COCONUT JAS are highlighted in green, fragment not exactly contained in the COCONUT JAS are highlighted in red. The most similar fragment are shown in the figure (Tanimoto similarity of Morgan fingerprints r=2, size=2048).

References

1. Jirasek, M. *et al.* Investigating and Quantifying Molecular Complexity Using Assembly Theory and Spectroscopy. *ACS Cent. Sci.* **10**, 1054–1064 (2024).
2. Greg Landrum *et al.* rdkit/rdkit: 2024_03_3 (Q1 2024) Release. Zenodo https://doi.org/10.5281/ZENODO.591637 (2024).
3. Morgan, H. L. The Generation of a Unique Machine Description for Chemical Structures-A Technique Developed at Chemical Abstracts Service. *J. Chem. Doc.* **5**, 107–113 (1965).

4. Gugisch, R. *et al.* Chapter 6 - MOLGEN 5.0, A Molecular Structure Generator. in *Advances in Mathematical Chemistry and Applications* (eds Basak, S. C., Restrepo, G. & Villaveces, J. L.) 113–138 (Bentham Science Publishers, 2015). doi:10.1016/B978-1-68108-198-4.50006-0.
5. Rappe, A. K., Casewit, C. J., Colwell, K. S., Goddard, W. A. & Skiff, W. M. UFF, a full periodic table force field for molecular mechanics and molecular dynamics simulations. *J. Am. Chem. Soc.* **114**, 10024–10035 (1992).
6. Wang, S., Witek, J., Landrum, G. A. & Riniker, S. Improving Conformer Generation for Small Rings and Macrocycles Based on Distance Geometry and Experimental Torsional-Angle Preferences. *J. Chem. Inf. Model.* **60**, 2044–2058 (2020).
7. Lim, V. T., Hahn, D. F., Tresadern, G., Bayly, C. I. & Mobley, D. L. Benchmark assessment of molecular geometries and energies from small molecule force fields. *F1000Research* **9**, Chem Inf Sci-1390 (2020).
8. Pedregosa, F. *et al.* Scikit-learn: Machine Learning in Python. *J. Mach. Learn. Res.* **12**, 2825–2830 (2011).
9. Dice, L. R. Measures of the Amount of Ecologic Association Between Species. *Ecology* **26**, 297–302 (1945).
10. Baell, J. B. & Holloway, G. A. New Substructure Filters for Removal of Pan Assay Interference Compounds (PAINS) from Screening Libraries and for Their Exclusion in Bioassays. *J. Med. Chem.* **53**, 2719–2740 (2010).
11. Kim, S. *et al.* PubChem 2023 update. *Nucleic Acids Res.* **51**, D1373–D1380 (2023).
12. Sorokina, M., Merseburger, P., Rajan, K., Yirik, M. A. & Steinbeck, C. COCONUT online: Collection of Open Natural Products database. *J. Cheminformatics* **13**, 2 (2021).
13. Virtanen, P. *et al.* SciPy 1.0: fundamental algorithms for scientific computing in Python. *Nat. Methods* **17**, 261–272 (2020).

14. Klapper, M. *et al.* Natural products from reconstructed bacterial genomes of the Middle and Upper Paleolithic. *Science* **380**, 619–624 (2023).
